# Ganglionated Plexi Ablation Suppresses Chronic Obstructive Sleep Apnea-Related Atrial Fibrillation by Inhibiting Cardiac Autonomic Hyperactivation

**DOI:** 10.3389/fphys.2021.640295

**Published:** 2021-04-09

**Authors:** Ling Zhang, Yankai Guo, Jiasuoer Xiaokereti, Guiqiu Cao, Hongliang Li, Huaxin Sun, Kai Li, Xianhui Zhou, Baopeng Tang

**Affiliations:** ^1^Xinjiang Key Laboratory of Cardiac Electrophysiology and Cardiac Remodeling, The First Affiliated Hospital of Xinjiang Medical University, Urumqi, China; ^2^Cardiac Pacing and Electrophysiology Department, The First Affiliated Hospital of Xinjiang Medical University, Urumqi, China; ^3^Department of Cardiology, The Fifth Affiliated Hospital of Xinjiang Medical University, Urumqi, China; ^4^Section of Endocrinology and Diabetes, Department of Medicine, University of Oklahoma Health Sciences Center, Oklahoma City, OK, United States

**Keywords:** atrial fibrillation, obstructive sleep apnea, sympathovagal hyperactivity, ganglionated plexi (GP), ablation

## Abstract

**Background:** Previous studies have reported that right pulmonary artery ganglionated plexi (GP) ablation could suppress the onset of atrial fibrillation (AF) associated with obstructive sleep apnea (OSA) within 1 h.

**Objective:** This study aimed to investigate the effect of superior left GP (SLGP) ablation on AF in a chronic OSA canine model.

**Methods and Results:** Fifteen beagles were randomly divided into three groups: control group (CTRL), OSA group (OSA), and OSA + GP ablation group (OSA + GP). All animals were intubated under general anesthesia, and ventilation-apnea events were subsequently repeated 4 h/day and 6 days/week for 12 weeks to establish a chronic OSA model. SLGP were ablated at the end of 8 weeks. SLGP ablation could attenuate the atrial effective refractory period (ERP) reduction and decrease ERP dispersion, the window of vulnerability, and AF inducibility. In addition, chronic OSA leads to left atrial (LA) enlargement, decreased left ventricular (LV) ejection fraction, glycogen deposition, increased necrosis, and myocardial fibrosis. SLGP ablation reduced the LA size and ameliorated LV dysfunction, while myocardial fibrosis could not be reversed. Additionally, SLGP ablation mainly reduced sympathovagal hyperactivity and post-apnea blood pressure and heart rate increases and decreased the expression of neural growth factor (NGF), tyrosine hydroxylase (TH), and choline acetyltransferase (CHAT) in the LA and SLGP. After SLGP ablation, the nucleotide-binding oligomerization domain (NOD)-like receptor signaling pathway, cholesterol metabolism pathway, and ferroptosis pathway were notably downregulated compared with OSA.

**Conclusions:** SLGP ablation suppressed AF in a chronic OSA model by sympathovagal hyperactivity inhibition. However, there were no significant changes in myocardial fibrosis.

## Introduction

Obstructive sleep apnea (OSA) is one of the most common forms of sleep breathing disorders and may affect ~24% of men and 9% of women between 30 and 60 years of age (Calkins et al., 2017). A previous study clearly identified that patients with OSA have a 4-fold risk of developing atrial fibrillation (AF) (Mehra et al., 2006), a higher risk of AF recurrence after cardioversion and catheter ablation (Kanagala et al., 2003; Tang et al., 2009; Fein et al., 2013), and a weak response to anti-arrhythmic drugs (Matiello et al., 2010). Therefore, it is an urgent problem to further explore an effective treatment for patients with OSA-associated AF.

The role of ganglionated plexi (GP), known as the intrinsic cardiac autonomic nervous system, containing abundant nerve axons, interconnected neurons, and autonomic ganglia clusters, embedded into the epicardial fat pads, has been increasingly recognized (Stavrakis and Po, 2017). Several studies have reported that hyperactivity of the ganglionated plexi (GP) promotes the initiation and maintenance of AF, and both animal (Hou et al., 2007; Lu et al., 2008; Ghias et al., 2009; Yu et al., 2017c) and clinical studies (Scanavacca et al., 2006; Katritsis et al., 2008; Po et al., 2009) have shown that AF can be inhibited by GP ablation. Ghias et al. (2009) reported that right pulmonary artery GP ablation could decrease 1-h apnea-induced AF by suppressing anterior right ganglionated plexus (ARGP) activity. Yu et al. (2017c) also revealed that hyperactivity of superior left GP (SLGP) promoted the initiation and maintenance of AF, and AF inducibility could be suppressed by low-level transcutaneous electrical stimulation (LLTS) in an acute intermittent hypoxia model in dogs. All of these studies demonstrated that GP inhibition/ablation could suppress acute OSA-induced AF by inhibiting the hyperactivity of the autonomic nervous system (ANS).

However, the long-term effects of GP ablation on chronic OSA-induced AF and the underlying mechanisms of AF suppression have not yet been clearly elucidated. We hypothesized that GP ablation could suppress chronic OSA-related AF by inhibiting cardiac autonomic hyperactivation. In our current study, we constructed a chronic OSA model in canines and explored the long-term effects and underlying mechanisms of SLGP ablation on the development and progression of AF.

## Methods

All animal protocols were approved by the Ethics Committee of Animal Experiments of the First Affiliated Hospital of Xinjiang Medical University (permit number: IACUC201902-K04), which strictly complied with the requirements in the Guide for the Care and Use of Laboratory Animals of the National Institutes of Health (NIH Publication No. 85-23, revised 1996). All animals were provided by Nanjing Yadong Experimental Animal Research Center [permit number: SCXK (SU): 2013-0001].

### Animal Preparation

Fifteen adult male beagles weighing 15–18 kg were investigated in this study. Intramuscular injection of Zoletil (0.1 mg/kg, Virbac SA, France) and xylazine (5 mg/kg, Huamu Animal Health Care Products Co., Ltd., China) was used to induce anesthesia. After the eyelash reflex disappeared, the trachea was intubated. During the experiment, 3% sodium pentobarbital solution was continuously pumped intravenously at 2 ml/h to maintain anesthesia.

### Establishment of the OSA Model

**Figure 1:**
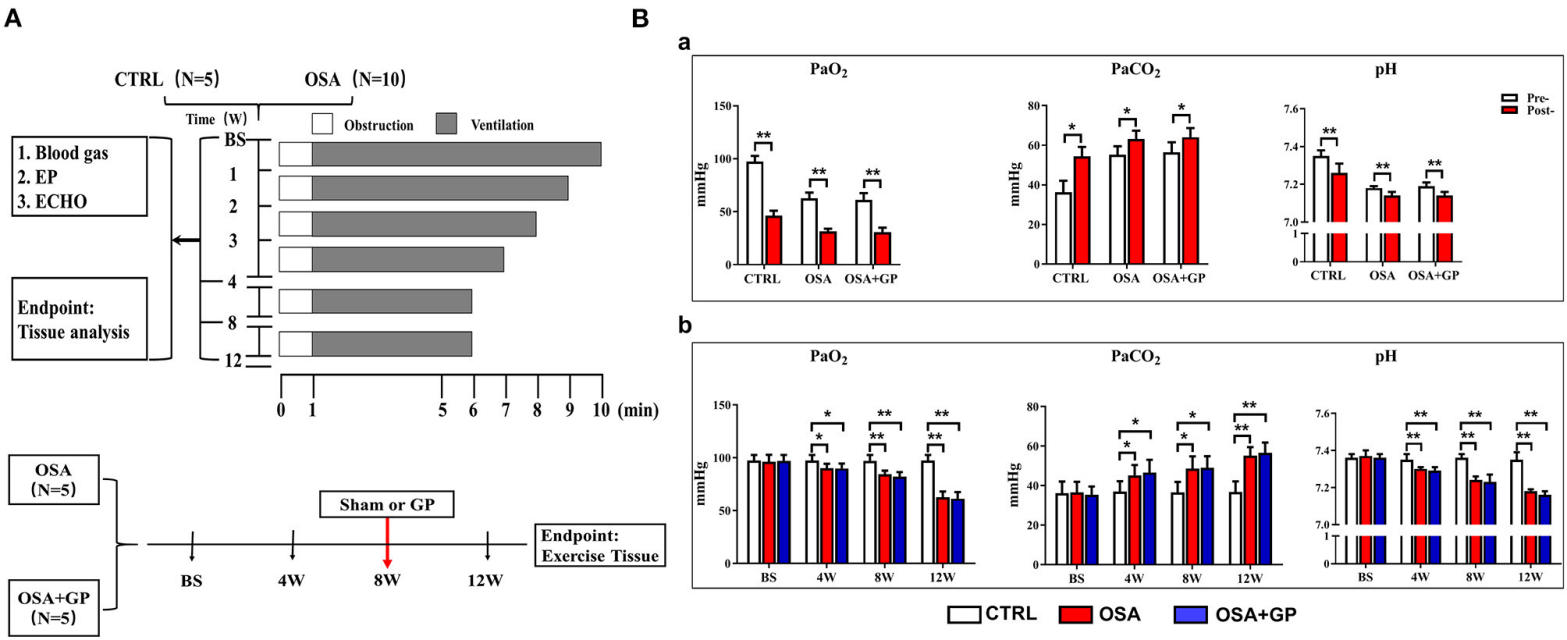
**(A)** Study protocol performed on the dogs. **(B)** Changes in blood gas among the three groups. (a) After 1 min of apnea in the 12th week of simulating OSA; (b) During the 12-week period of OSA simulation before apnea. *P < 0.05, **P < 0.01, *n* = 5. OSA, obstructive sleep apnea; GP, ganglionated plexus; CTRL, control; BS, baseline; EP, electrophysiology; ECHO, echocardiography; Pre-, pre-apnea; Post-, post-apnea; W, week.

The OSA model was established by blocking the endotracheal cannula at the end of exhalation, as described previously (Zhao et al., 2014; Gao et al., 2015). From the 1st week to the 5th week, the spontaneous breathing time decreased weekly from 9 to 5 min and was maintained for 5 min until the end of the experiment. The apnea time always lasted 1 min. This breath–sleep apnea cycle event was repeated for 4 h/day, 6 days/week, for a total of 12 weeks (Figure 1A).

### Experiment Protocol

Fifteen beagles were randomly divided into three groups: OSA (OSA for 12 weeks with sham GP ablation, *n* = 5); OSA + GP (OSA for 12 weeks, GP ablation was performed at the end of the 8th week through left thoracotomy at the fourth intercostal space, *n* = 5); and CTRL (sham OSA without GP ablation, *n* = 5). For the CTRL, we gave intubation and anesthesia 4 h a day without blocking the endotracheal cannula and GP ablation. Figure 1A illustrates the protocol.

### Blood Gases

Blood gases were determined at baseline and at the 4th, 8th, and 12th week before 1 min of apnea. At the same time, after 1 min of apnea in the 12th week of OSA simulation among the three groups, blood gases were also determined. Oxygen partial pressure (PaO_2_), carbon dioxide pressure (PaCO_2_), and pH were detected within 1 min once the sample was drawn with an i-STAT300 Analyzer (Abbott Laboratories, USA).

### Programmed Stimulation

At baseline and at the 4th, 8th, and 12th week, the femoral artery and vein were cannulated. Standard limb lead electrocardiograms, intracardiac electrophysiological recordings, and arterial blood pressure (BP) were continuously monitored (LEAD 7000, Jinjiang Inc., Chengdu, China) throughout the experiment. The SLGP was ablated at the end of the 8th week through left thoracotomy at the fourth intercostal space.

The atrial effective refractory period (ERP), ERP dispersion (dERP), AF inducibility, AF duration, sinus node recovery time (SNRT), and window of vulnerability (WOV) were determined at baseline and at the 4th, 8th, and 12th week. Programmed stimulation at the high right atrium (HRA) was administered with eight consecutive stimuli (S1–S1 cycle length 330 ms) followed by premature stimuli (S1–S2) at a 4-fold threshold. The S1 to S2 interval gradually decreased from 180 ms initially in decrements of 10–5 ms until capture no longer occurred. Burst stimulation was performed with consecutive bursts of rapid atrial pacing (S1-S1 cycle length 50 ms, 8 V, lasting 10 s). AF was defined as irregular atrial beats (≥500 bpm, lasting ≥5 s) with irregular atrioventricular (AV) conduction (Gao et al., 2015; Yu et al., 2017b). The WOV, defined as the difference between the maximum and minimum S1–S2 intervals that induced AF, was adopted as an indicator of AF inducibility (Gao et al., 2015; Yu et al., 2017b). The dERP was defined as the difference between the longest and the shortest value of the ERP at six recording sites, including HRA, left atrial appendage (LAA), right superior pulmonary vein (RSPV), right inferior pulmonary vein (RIPV), left superior pulmonary vein (LSPV), and left inferior pulmonary vein (LIPV).

### SLGP Location and Ablation

**Figure 2:**
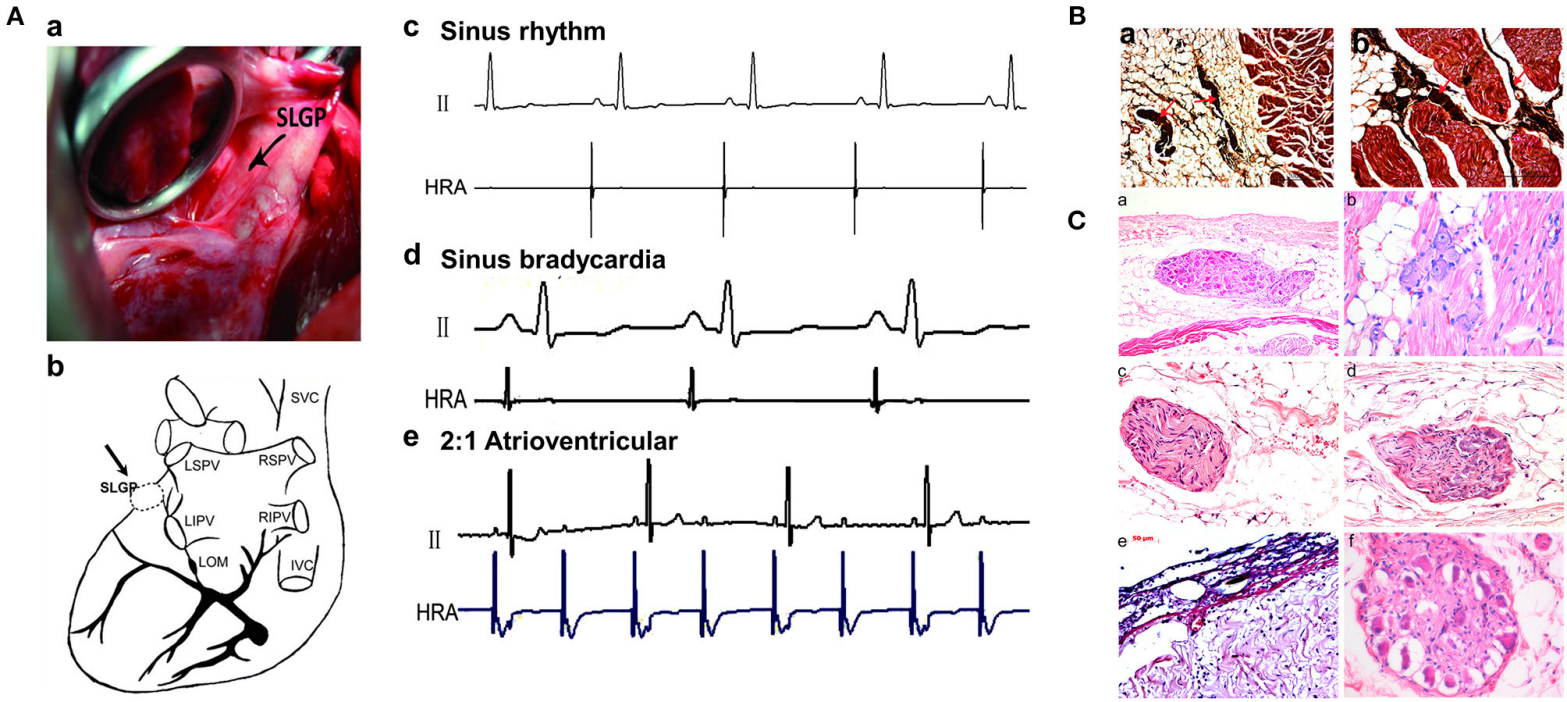
Changes in SLGP before and after ablation. **(A)** Location of the SLGP. The place marked with an arrow is the position of the SLGP (a,b); before and after SLGP stimulation, different types of arrhythmias were observed (c–e). **(B)** Silver staining of the SLGP. **(C)** Representative images of HE staining for SLGP: (a) dozens of neurons in SLGP were observed in subepicardial fat tissue; (b) several neurons were observed between cardiac muscle; (c) The nerve fibers and neurons in a nerve bundle; (d) the nerve fiber bundle; (e) disorder of subepicardial collagen tissue structure after SLGP ablation; (f) ganglion cells developed vacuolar degeneration after SLGP ablation. SVC, superior vena cava; IVC, inferior vena cava; RSPV, right superior pulmonary vein; RIPV, right inferior pulmonary vein; LSPV, left superior pulmonary vein; LIPV, left inferior pulmonary vein; SLGP, superior left ganglionated plexus; LOM, ligament of Marshall; HRA, high right atrium; HE, hematoxylin and eosin; other abbreviations as in Figure 1.

Thoracotomy was performed at the left fourth intercostal space to expose the pericardium, and then the SLGP was found to be located adjacent to the LSPV–LA junction between the LAA and left pulmonary artery (Figures 2Aa,b). High-frequency stimulation (20 Hz, 0.1 ms pulse width, 0.6–4.5 V) (Grass S88, Astro-Med Inc., West Warwick, RI, USA) was administered until the R–R interval increased by 50% or a 2:1 atrioventricular block emerged, indicating that the position of the SLGP was correct (Po et al., 2009). Ablation was performed using an irrigated large-tip (3.5 mm) electrode catheter (Biosense-Webster Inc. Diamond Bar, CA, USA). During ablation energy delivery, marked slowing of the sinus rate and/or AV conduction was observed (Cooper et al., 1980; Schauerte et al., 2000). When applying 12 V to stimulate the SLGP with an electrode catheter after energy delivery, the absence of slowing of the sinus rate and/or AV conduction indicated that the ablation was complete (Lu et al., 2010) (Figures 2Ac–e).

### Echocardiography

Echocardiographic examination was conducted with Doppler echocardiography (Sonos 5500, Philips Ultrasound, USA) at a 3.5-MHz ultrasound probe, and transthoracic 2-dimensional (2D) and M-mode imaging were performed at baseline and at the 4th, 8th, and 12th week. Right atrial end-diastolic diameter (RAd), left atrial end-diastolic diameter (LAd), left ventricular end-diastolic diameter (LVEDD), and left ventricular ejection fraction (LVEF) were determined. Measurements were averaged over three to five consecutive cardiac cycles, and mean values were used for statistical analysis.

### Heart Rate Variability Analysis

Five minutes of ECG recordings were obtained when the dog was awake and quiet to perform heart rate variability (HRV) analysis to reveal changes in ANS activity. A data acquisition system (LabChart Pro, AD Instruments Ltd) was used to analyze its frequency domain indicators: low-frequency power (LF, 0.04–0.15 Hz, assumed to have a dominant sympathetic component), high-frequency power (HF, 0.15–0.40 Hz, reflecting cardiac parasympathetic nerve activity), and the low-frequency-to-high-frequency power ratio (LF/HF ratio, quantifying the changing relationship between sympathetic and parasympathetic nerve activities). Three consecutive measurements were averaged for each variable.

### Neural Activity Recording

Neural activities of the left vagal nerve (LVN) and left stellate ganglion (LSG) were recorded among the three groups at the 12th week, as described previously (Zhou et al., 2016; Zhang et al., 2018). All the animals were anesthetized during the neural activity recordings. Briefly, the LVN and LSG were exposed via a left vertical incision in the supraclavicular fossa and bluntly dissected free from surrounding tissues with a glass dissecting needle. Neural activities were recorded through headstage electrodes into the LVN and SLGP, and nerve signals were analyzed with the Analysis Module of Lab Chart 8.0/proV7 software (Bio Amp; ADInstruments) of PowerLab (Bio Amp; ADInstruments). An amplifier (DP-304; Warner Instruments) was used to amplify nerve signals.

### Histological Study

The SLGP [located at the roof of the left atrium, 1–2 cm medial to the left superior PV (Stavrakis and Po, 2017)], LVN, LSG, and myocardial tissues (SLGP, LA, LAA, RAA, LV, and RV) were collected and fixed in paraformaldehyde after euthanasia. All tissues were embedded in paraffin and cut into 4-μm-thick sections. Then, hematoxylin and eosin (HE) staining, Masson's trichrome staining, periodic acid-Schiff (PAS) staining, terminal deoxynucleotidyl transferase dUTP nick end labeling (TUNEL) staining, and silver staining were performed according to the manufacturer's instructions (Western Biomedical Technology, Hubei, China). A charge-coupled device (CCD) camera attached to an inverted microscope (Zeiss, Germany) was used to capture the image of the stained cells. Image analysis software (Image-Pro Plus: IPP 7.0, Media Cybernetics LP) was used to estimate the area of interstitial fibrosis, glycogen volume fraction of the tissues, and apoptotic rate of cardiomyocytes.

### Immunohistochemistry

Tissues sections of the LVN, LSG, SLGP, and LA that were paraffin-embedded were used to perform immunohistochemistry (IHC). The avidin–biotin complex method was performed to detect the expression levels of the following proteins: TH (tyrosine hydroxylase, LS C354110, 1:100, LifeSpan BioSciences, Seattle, WA, USA); CHAT (choline acetyltransferase, LS C79271, 1:100, LifeSpan BioSciences, Seattle, WA, USA); NGF (neural growth factor, LS C388946, 1:100 dilution, LifeSpan BioSciences, Seattle, WA, USA); connexin-40 (Cx40, LS B959, 1:100, LifeSpan BioSciences, Seattle, WA, USA); and connexin-43 (Cx43, LS B9771, 1:100, LifeSpan BioSciences, Seattle, WA, USA). Slices were incubated with different antibodies overnight at 4°C separately and subsequently incubated with peroxidase-conjugated rat anti-rabbit IgG (LS-C60921, LifeSpan BioSciences, Seattle, WA, USA) at 37°C for 20 min. Finally, a microscope (Leica, Wetzlar, Germany) was used to evaluate the samples. The images were analyzed with Image-Pro Plus 6.0 software (Media Cybernetics, USA).

### Proteomics Analysis

Frozen tissue was ground in liquid nitrogen into a cell powder, and lysis buffer was added to extract the protein, followed by trypsin digestion and TMT/iTRAQ labeling. Then, an EASY-nLC 1000 UPLC system was used to analyze the expression of each peptide. The resulting tandem mass spectrometry (MS/MS) data were processed using the MaxQuant search engine (v.1.5.2.8) and Paragon protein database. Finally, the Kyoto Encyclopedia of Genes and Genomes (KEGG) database was used to identify enriched pathways.

### Statistical Analysis

Data are presented as the mean ± SD. Three group comparisons were performed via two-way ANOVA followed by Tukey's tests. Analysis of variance with *post-hoc* Tukey's test was used to compare continuous variables among different time points of apnea. A paired *t*-test was used for comparisons of BP, HR, and blood gases values before and after apnea was induced. A two-tailed Fisher's exact test was used to test the enrichment of the differentially expressed proteins against all identified proteins. SPSS 19.0 (IBM Corp.) was used for statistical analysis. GraphPad Prism 6.0 was used to draw all the graphics. Statistical significance was set at *P* < 0.05.

## Results

### Blood Gases

As shown in Figure 1Ba, after 1 min of apnea in the 12th week of OSA simulation, PaO_2_ and pH were significantly decreased, while PaCO_2_ was markedly increased (*P* < 0.05). Moreover, artery blood gases in pre-apnea were also detected at baseline and at the 4th, 8th, and 12th week, and no statistical significance in terms of pH, PaCO_2_, and PaO_2_ was shown among the three groups at baseline (*P* > 0.05). As the experiment progressed, PaO_2_ and pH decreased gradually, while PaCO_2_ increased notably (*P* < 0.05) (Figure 1Bb).

In short, during the early and later period of OSA, hypoxemia, hypercapnia, and acidosis manifested, while SLGP ablation did not alter this hypoxemic environment.

### Changes in SLGP Structure Before and After GP Ablation

As shown in Figures 2B,C, the structure of the SLGP was shown with silver and HE staining, indicating that the nerve bundle was located in the fat tissue and connective tissue between the cardiac muscle (Figures 2Ba,b). Once the GP was ablated, disorder of the subepicardial collagen tissue structure and the development of ganglion cell vacuolar degeneration could be observed in the SLGP with HE staining (Figures 2Ca–f).

### SLGP Ablation Ameliorated Atrial Electrical Remodeling in Chronic OSA-Induced AF

**Figure 3:**
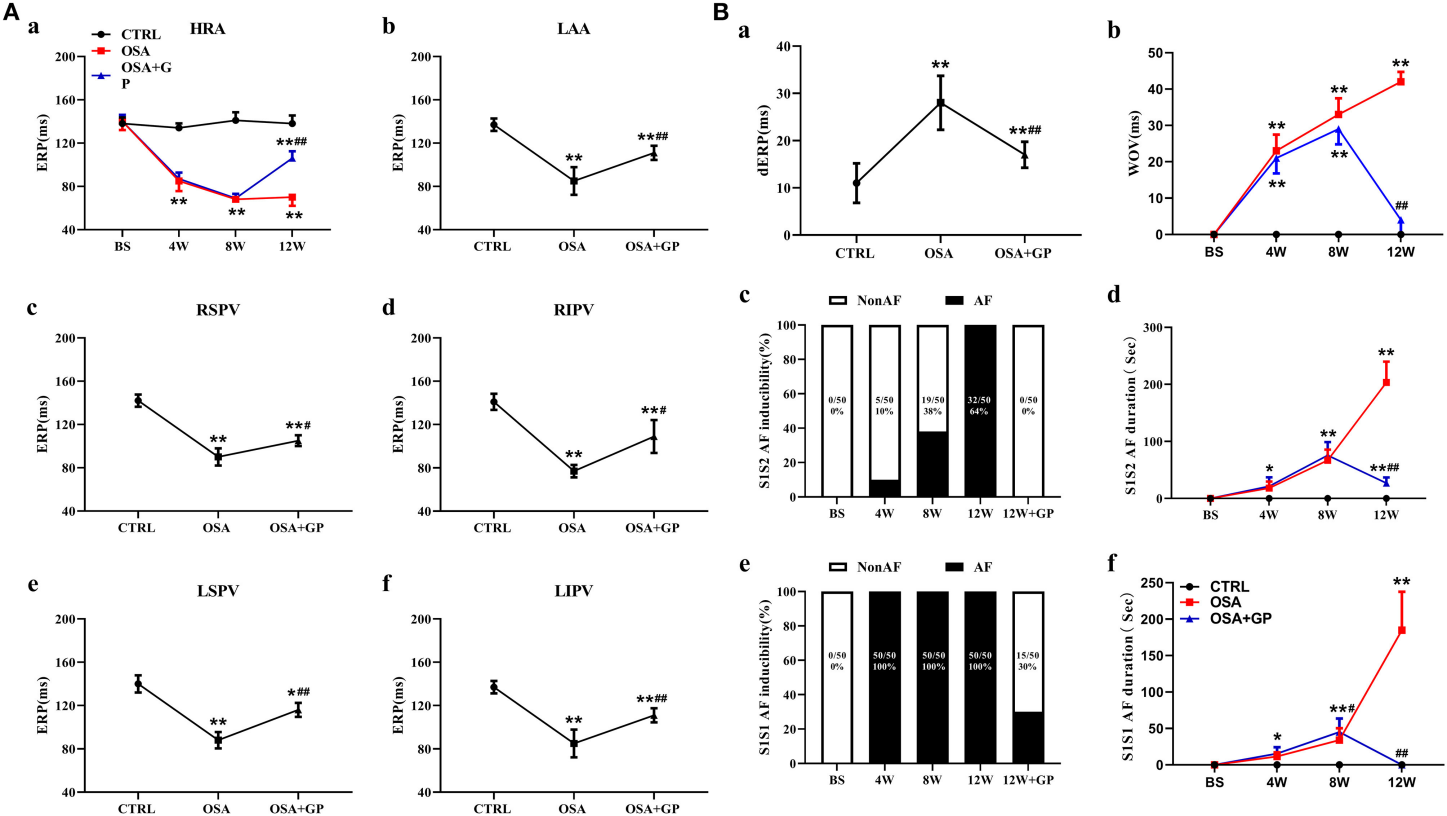
Effect of SLGP ablation on electrical remodeling. **(A)** Change in the mean ERP at different paced sites among the three groups (a–f). **(B)** Change in dERP (a) and WOV (b) among the three groups. The mean value of AF duration and AF inducibility (atrial tachyarrhythmia ≥5 s) after 10 S1–S2 pacing (c, d) and burst (S1–S1) pacing (e, f) events for each dog. *P < 0.05 vs. CTRL, **P < 0.01 vs. CTRL, ^#^*P* < 0.05 vs. OSA, ^##^*P* < 0.01 vs. OSA, *n* = 5. AF, atrial fibrillation; dERP, effective refractory period dispersion; WOV, window of vulnerability; other abbreviations as in Figures 1, 2.

As shown in Figure 3A, the ERP of the HRA (Figure 3Aa) was recorded from the beginning to the end of the study. No difference in the ERP value was observed among the three groups at baseline (*P* > 0.05). With the progression of OSA, ERP was gradually and significantly shortened at the 4th and 8th week in the OSA group and the OSA + GP group compared to the CTRL group (*P* < 0.01). Four weeks after SLGP ablation (week 12), ERP in the OSA + GP group gradually returned to the baseline level (*P* < 0.01). In addition, similar differences in ERP were also detected at all paced sites among the three groups at week 12. Compared with the CTRL group, the ERP value in the OSA group was obviously decreased, while the ablation of SLGP inhibited this effect (Figures 3Ab–f).

As shown in Figure 3Ba, the dERP in the OSA group was significantly increased compared to that in the CTRL group (*P* < 0.01) and was obviously decreased in the OSA + GP group. The WOV among the three groups was not significantly different at baseline (*P* > 0.05). As OSA was prolonged, the WOV increased notably in the OSA group and the OSA + GP group compared to the CTRL group at the 4th week and 8th week, and it was significantly decreased after 4 weeks of SLGP ablation (*P* < 0.01) (Figure 3Bb).

**Figure 4:**
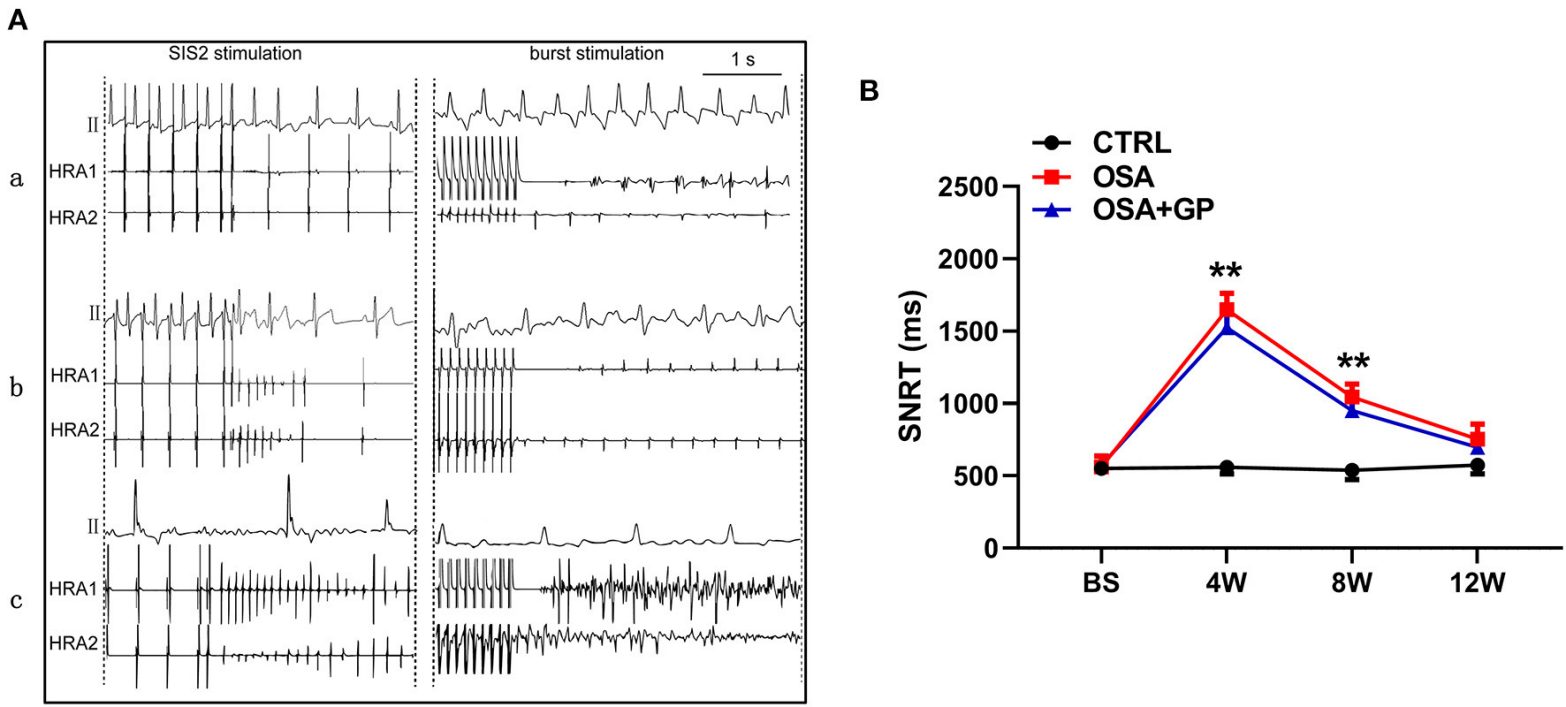
**(A)** Typical examples of AF episodes induced by OSA after S1–S2 and burst stimulation among the three groups; **(B)** Changes in SNRT among the three groups. **P < 0.01 vs. CTRL, *n* = 5. AF, atrial fibrillation; SNRT, sinus node recovery time; other abbreviations as in Figure 1.

Furthermore, with the progression of OSA, the AF duration and AF inducibility following S1–S2 stimulation in the OSA group gradually increased, and AF inducibility in the OSA group was as high as 100% at week 12, whereas AF could not be induced with S1–S2 stimulation in the OSA + GP group once the SLGP was ablated at week 8 (Figures 3Bc,d). In addition, AF duration and AF inducibility following S1–S1 burst stimulation in the OSA group were obviously increased, and the change tendency was similar to S1–S2 stimulation (Figures 3Be,f). The AF inducibility was 100% at week 4 until the end of week 12. After SLGP ablation, the AF inducibility via burst pacing in the OSA + GP group was <30%. A representative AF episode is shown in Figure 4A.

Additionally, SNRT was also analyzed among the three groups (Figure 4B). From baseline to the 4th week, the value of SNRT was markedly increased in the OSA and OSA + GP groups, and it gradually returned to baseline.

**Figure 5:**
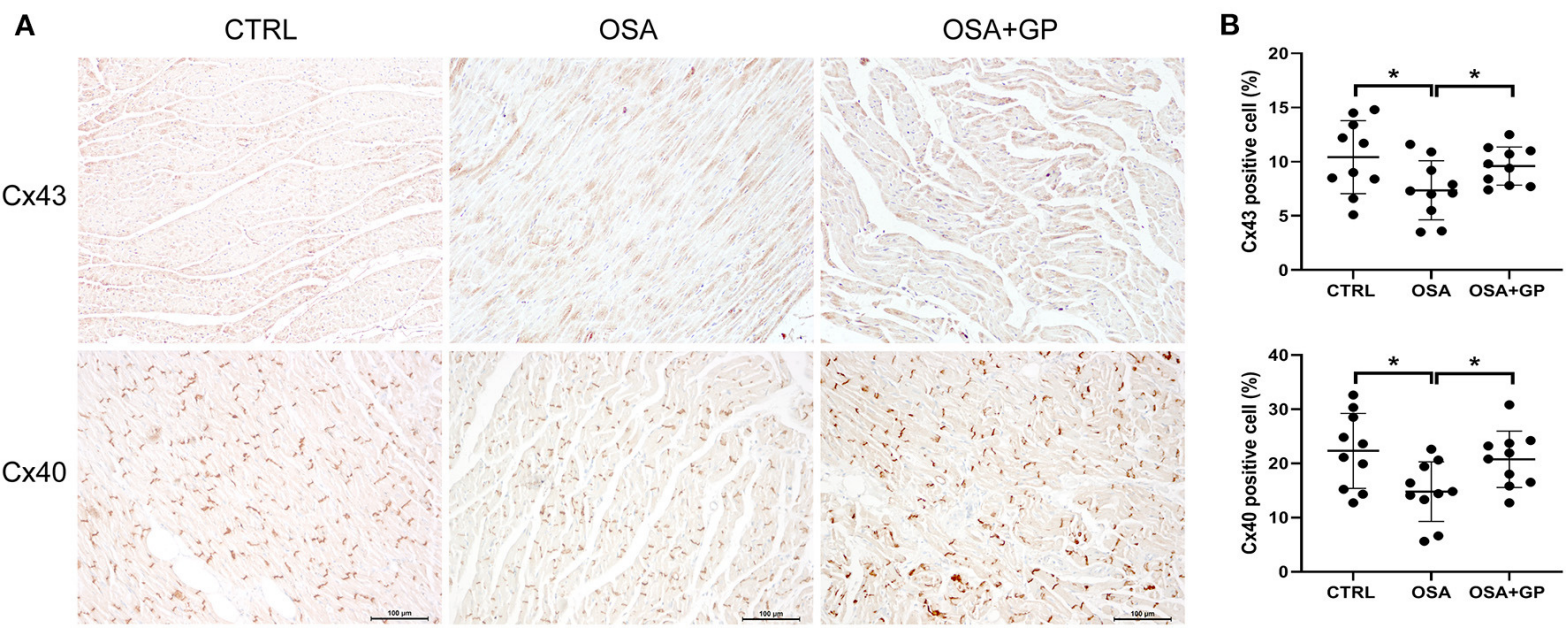
Effect of SLGP ablation on electrical conduction heterogeneity. **(A)** Representative immunohistochemical staining of Cx43 and Cx40 in LA among the three groups. **(B)** Quantitative analysis of Cx43- and Cx40-positive cells among the three groups. *P < 0.05, *n* = 5. Cx43, connexin 43; Cx40, connexin 40; other abbreviations as in Figure 1.

Furthermore, the expression levels of Cx43 and Cx40 were also determined with IHC (Figure 5). Compared with the CTRL group, the expression levels of Cx43 and Cx40 were decreased in the OSA group, while SLGP ablation increased their expression in the OSA + GP group.

In summary, the chronic OSA model is prone to increased AF inducibility, and the effect could be inhibited by SLGP ablation.

### Effects of SLGP Ablation on Structural Remodeling in Chronic OSA-Induced AF

**Figure 6:**
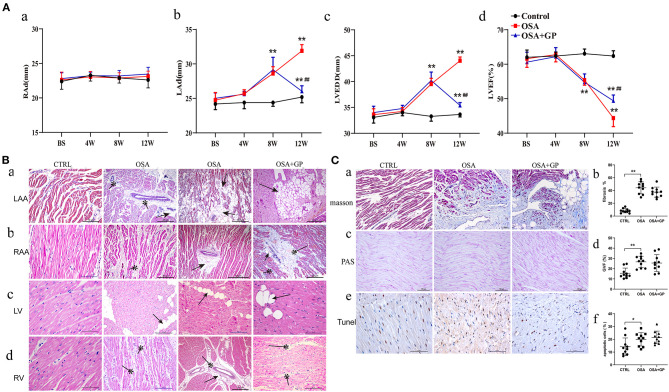
Effects of SLGP ablation on structural remodeling. **(A)** Changes of echocardiographic parameters among the three groups; **(B)** HE staining of typical myocardial histopathological changes. **(C)** Myocardial histopathological changes in LA were observed with different staining (a–c): quantitative analysis of Masson staining (b), PAS staining (d), and TUNEL staining (f) among the three groups. *P < 0.05 vs. CTRL, **P < 0.01 vs. CTRL, ^##^*P* < 0.01 vs. OSA. RAd, right atrial end-diastolic diameter; LAd, left atrial end-diastolic diameter; LVEDD, left ventricular end-diastolic diameter; LVEF, left ventricular ejection fraction. RAA, right atrial appendage; LV, left ventricular; RV, right ventricular; TUNEL, terminal deoxynucleotidyl transferase dUTP nick end labeling; PAS, periodic acid-Schiff; other abbreviations as in Figure 1.

As shown in Figure 6A, after 8 weeks of simulating OSA, the LAd and LVEDD in the OSA and OSA + GP groups were significantly increased compared to the CTRL group (*P* < 0.01), while the LVEF was significantly decreased (*P* < 0.01). Interestingly, 4 weeks after SLGP ablation, decreased LAd and LVEDD and increased LVEF were observed (all *P* < 0.01). In addition, no significant change in RAd was observed among the three groups.

To evaluate cardiac injury in chronic OSA, HE staining was performed (Figure 6B). Compared with the CTRL group, considerable fatty infiltration of the LAA, extensive fibrosis of the RAA, and mild fatty infiltration and fibrosis of the left ventricle (LV) and RV were notably observed in the OSA group. SLGP ablation did not attenuate this structural remodeling within 4 weeks.

Because obvious changes were manifested in the LAA, Masson staining, PAS staining, and TUNEL staining were further conducted in LA tissues (Figure 6C). Cardiomyocyte fibrosis, glycogen deposition, and apoptosis were present in the OSA group, and SLGP ablation failed to reverse this negative effect.

In summary, the above results indicated that OSA caused cardiac dysfunction and cardiac remodeling. SLGP ablation treatment ameliorated cardiac dysfunction, but failed to reverse structural remodeling effects within 4 weeks.

### Effects of SLGP Ablation on the ANS in Chronic OSA-Induced AF

**Figure 7:**
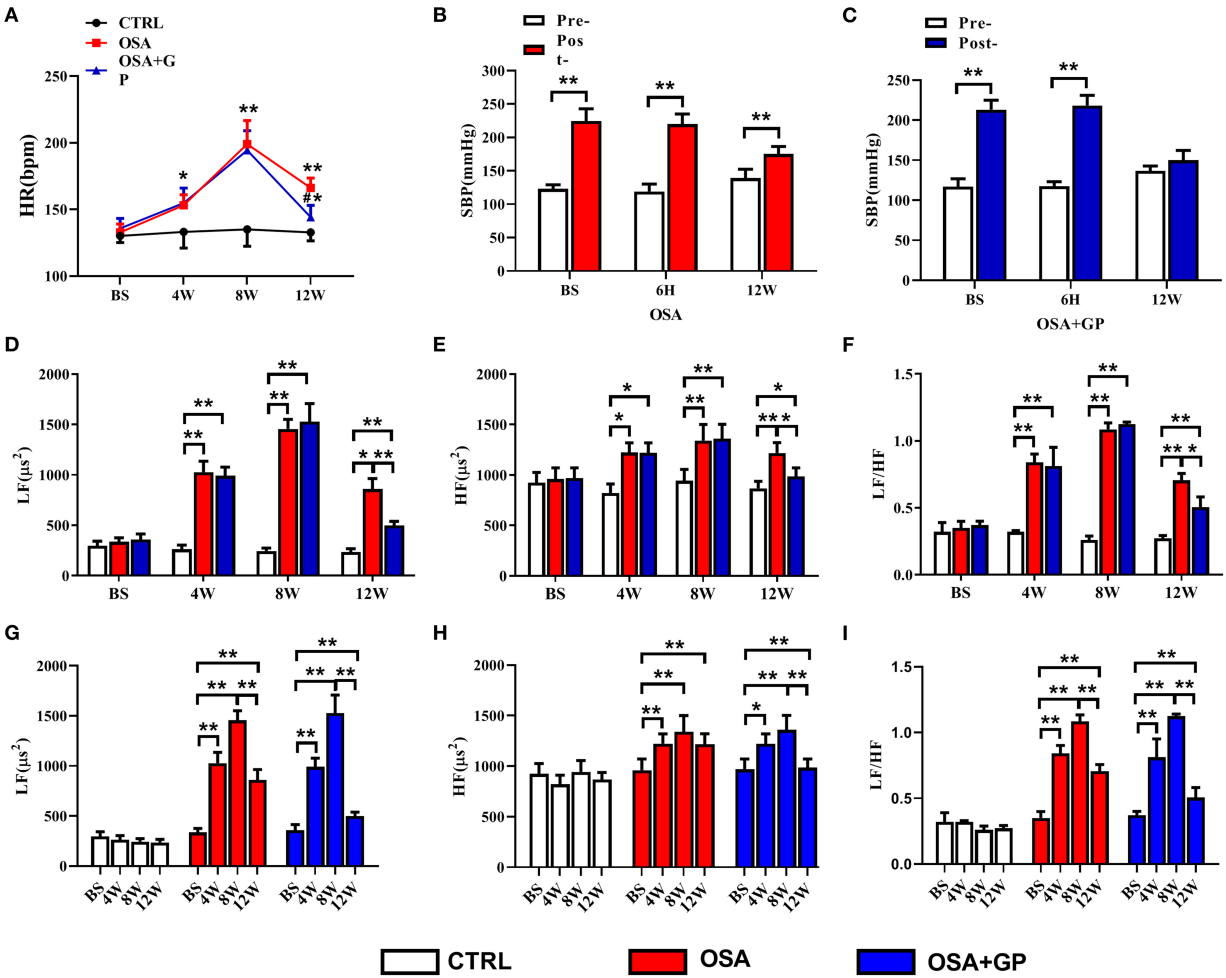
Effect of SLGP ablation on the HRV. **(A)** Changes in HR among the three groups, *P < 0.05 vs. CTRL, **P < 0.01 vs. CTRL, ^#^*P* < 0.05 vs. OSA. Changes in SBP in the OSA group **(B)** and the OSA + GP group **(C)** at baseline, 6 h, and the 12th week, **P < 0.01 vs. Pre-. Change in LF **(D)**, HF **(E)**, and LF/HF **(F)** among the three groups at baseline and the 4th, 8th, and 12th week, *P < 0.05, **P < 0.01. Change in LF **(G)**, HF **(H)**, and LF/HF **(I)** in each group at baseline and the 4th, 8th, and 12th week; *P < 0.05, **P < 0.01. HR, heart rate; SBP, systolic blood pressure; LF, low frequency; HF, high frequency; LF/HF, the ratio between LF and HF; other abbreviations as in Figure 1.

As shown in Figure 7A, with the progression of OSA, HR in the OSA and OSA + GP groups was significantly increased, reaching a maximum at the 8th week, and there was no statistically significant difference between the OSA and OSA + GP groups. At week 12, HR in the OSA group decreased, and the scale of the decrease was larger in the OSA + GP group (*P* < 0.05).

At the same time, changes in systolic blood pressure (SBP) in the OSA and OSA + GP groups at baseline (BS), the 6th h, and the 12th week were also detected. After 1 min of apnea, SBP in the OSA group dramatically increased, while the increased value in week 12 was lower than that at BS and 6 h (*P* < 0.01) (Figure 7B). In the OSA + GP group, the post-SBP at BS and 6 h was also increased, but no significant difference was observed in the 12th week (Figure 7C).

As shown in Figures 7D–I, LF, HF, and the LF/HF ratio were not significantly changed at baseline. With the progression of OSA, LF, HF, and the LF/HF ratio were significantly increased in the OSA group, and the scale of change in LF was much larger than that in HF. In addition, after GP ablation was administered at the end of the 8th week, LF, HF, and the LF/HF ratio were decreased, and HF decreased more. Compared with the 8th week, LF decreased 41.1% at the 12th week in the OSA group, and 67.3% in the OSA + GP group, so compared with the OSA group, LF decreased 1.64-fold in the OSA + GP group; similarly, compared with the OSA group, HF and LF/HF ratio in the OSA + GP group decreased 2.98 and 1.57-fold, respectively.

**Figure 8:**
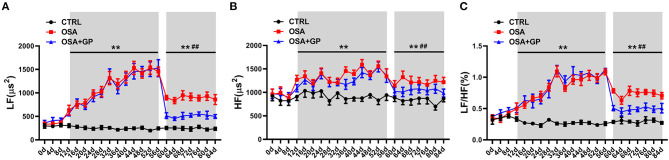
Dynamic consecutive changes in LF **(A)**, HF **(B)**, and LF/HF **(C)** among the three groups at baseline and the 4th, 8th, and 12th week, **P < 0.01 vs. CTRL, ^##^*P* < 0.01 vs. OSA; abbreviations as in Figures 1, 7.

Additionally, we observed consecutive changes in HRV every 4 days throughout the experiment (Figures 8A–C). At the beginning of 12 days, there was no significant change in LF, HF, or the LF/HF ratio. From 12 to 56 days, higher LF, HF, and LF/HF ratios were observed in the OSA group and the OSA + GP group than in the CTRL group. From 56 to 84 days, the values of LF, HF, and the LF/HF ratio in the OSA group and the OSA + GP group were decreased, and the scale of the decrease was larger in the OSA + GP group.

**Figure 9:**
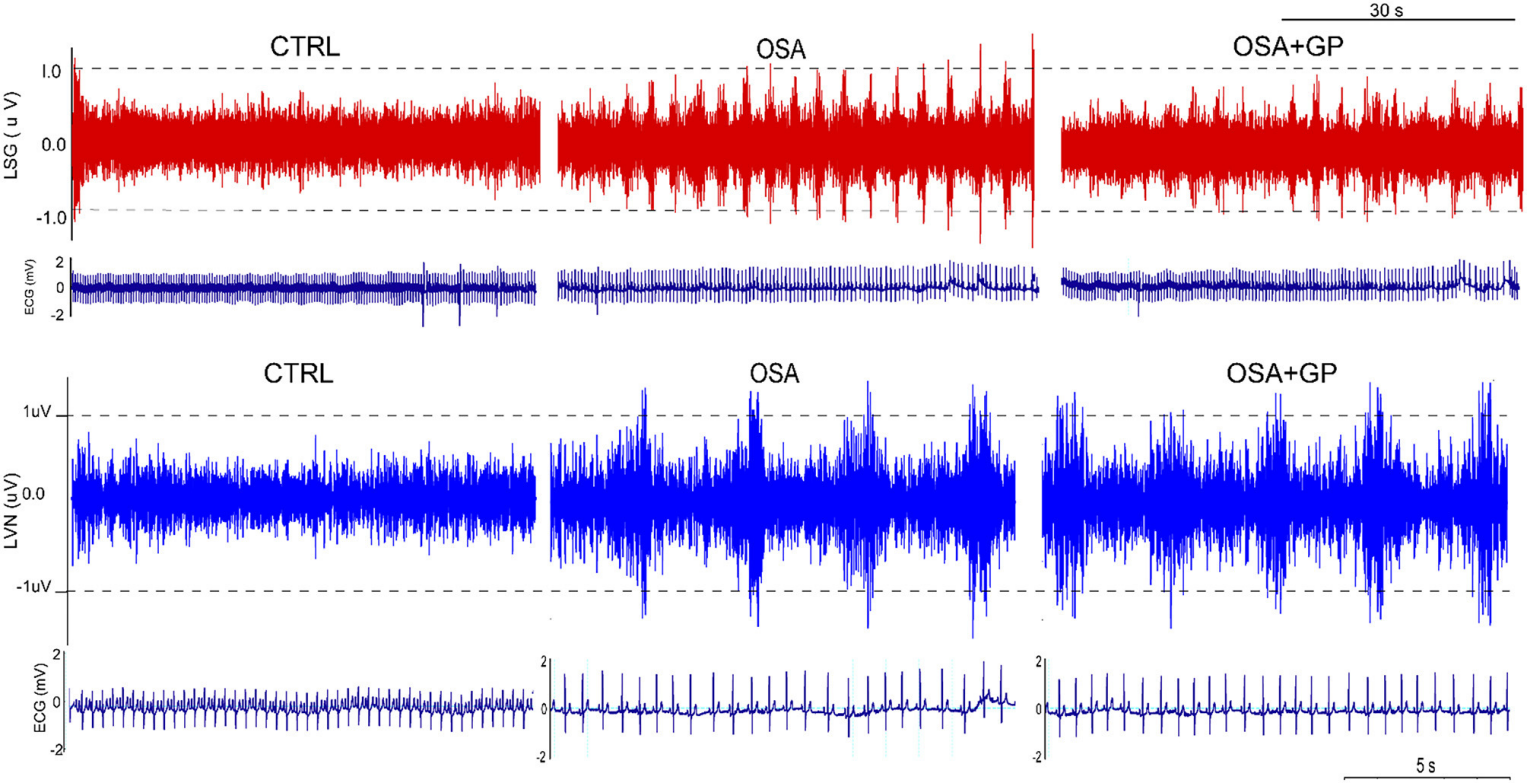
Effect of GP ablation on the neural activity. Representative examples of neural recordings from the LSG and LVN in different groups at the 12th week. LVN, left vagal nerve; LSG, left stellate ganglion; other abbreviations as in Figure 1.

As shown in Figure 9, neural activity of the LVN and LSG was significantly increased after simulating OSA at the 12th week in the OSA group compared to the CTRL group. However, the hyperactivity of the LVN and LSG was notably inhibited in the OSA + GP group compared to the OSA group.

**Figure 10:**
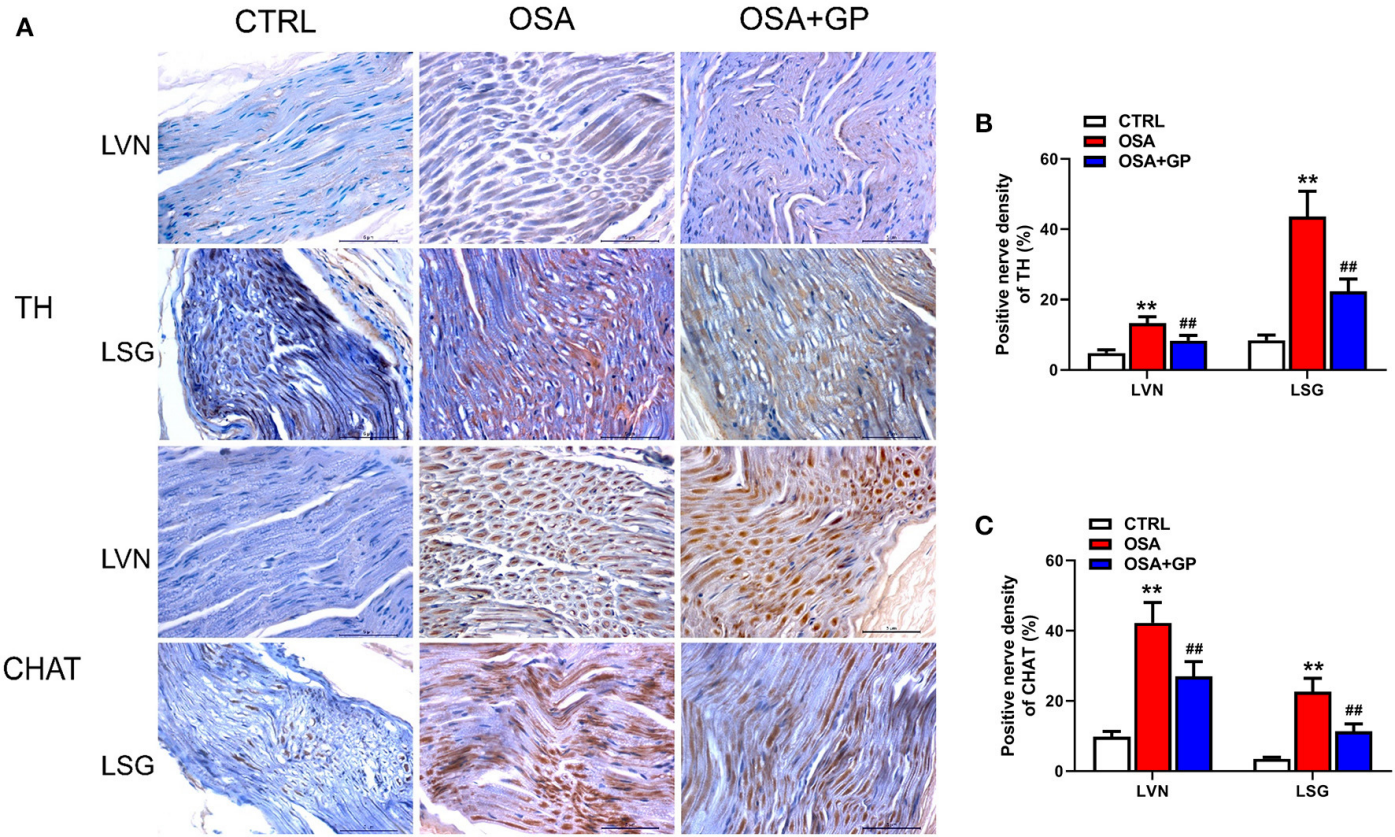
Effect of SLGP ablation on the expression level of TH and CHAT among the three groups. Representative immunohistochemical staining **(A)** and quantitative analysis of TH **(B)** and CHAT **(C)** in LVN and LSG; **P < 0.01 vs. CTRL, ^*##*^*P* < 0.01 vs. OSA; TH, tyrosine hydroxylase; CHAT, choline acetyltransferase; abbreviations as in Figures 1, 9.

**Figure 11:**
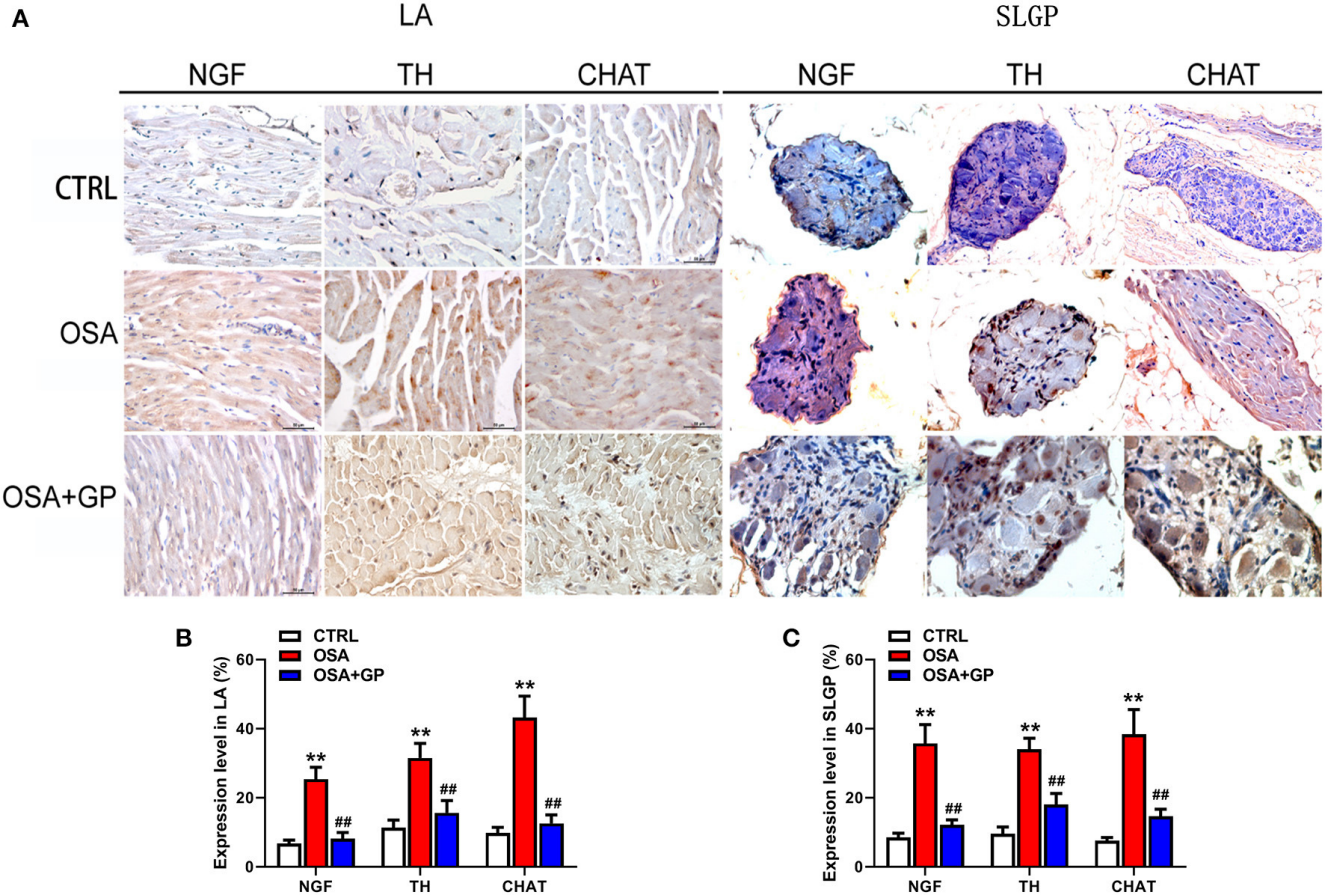
Effect of SLGP ablation on the expression level of NGF, TH, and CHAT among the three groups. Representative immunohistochemical staining **(A)** and quantitative analysis of NGF, TH, and CHAT in LA **(B)** and SLGP **(C)**; **P < 0.01 vs. CTRL, ^*##*^*P* < 0.01 vs. OSA. NGF, neural growth factor; LA, left atrial; other abbreviations as in Figures 1, 10.

Furthermore, neural indicators that could reflect the activity of the ANS were also detected by IHC. As shown in Figures 10, 11, the expression levels of NGF, TH, and CHAT were enhanced in the LA, SLGP, LVN, and LSG in the OSA group, and all of them were decreased in the OSA + GP group.

In summary, in the dog model of chronic OSA-induced AF, the activity of the ANS manifested a dynamic change, and the hyperactivity of the ANS could be inhibited by SLGP ablation.

### Proteomics Analysis

**Figure 12:**
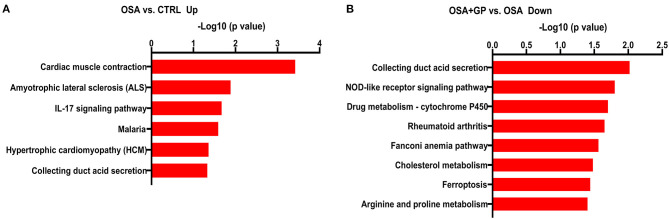
The *X*-axis represents the –log10 (*p*-value) showing the overrepresented pathways according to the identified differentially expressed genes for **(A)** OSA vs. CTRL (up expression) and **(B)** OSA + GP vs. OSA (down expression). The supplement that lists the specific genes for each pathway is also shown (*n* = 3 animals per intervention); abbreviations as in Figure 1.

The results of proteomics analysis are shown in Figure 12. Statistically significant differences were manifested between the OSA and CTRL groups in the interleukin (IL)-17 signaling pathway and indicate its role in autoimmune inflammatory disorders (Figure 12A). Moreover, after SLGP was ablated, the OSA + GP group (when compared with the OSA group) showed that the nucleotide-binding oligomerization domain (NOD)-like receptor signaling pathway was notably downregulated. The NOD-like receptors are responsible for detecting specific pathogen-associated molecules or host-derived damage signals in the cytosol and initiating the innate immune response. Also, cholesterol metabolism pathway and ferroptosis pathway were notably downregulated (Figure 12B). The specific genes are listed in the Supplementary Data.

## Discussion

### Major Findings

To the best of our knowledge, our study is the first to report that SLGP ablation inhibits electrical and neural remodeling in chronic OSA-associated AF in canines. We mainly found that (1) from baseline to the 12th week, ERP was gradually shortened and the WOV, AF inducibility, and AF duration were significantly increased, while SLGP ablation attenuated this effect and (2) chronic OSA led to left atrial (LA) enlargement, left ventricular (LV) ejection fraction decrease, glycogen deposition, increased necrosis, and myocardial fibrosis. SLGP ablation reduced the LA size and ameliorated LV dysfunction, while myocardial fibrosis could not be reversed. Additionally, SLGP ablation mainly reduced sympathovagal hyperactivity and the post-apnea blood pressure and heart rate increases and decreased the expression of NGF, TH, and CHAT in LA.

Our data suggested that SLGP ablation suppresses chronic obstructive sleep apnea-related atrial fibrillation by inhibiting cardiac autonomic hyperactivity, not by reversing myocardial structural remodeling.

### SLGP Ablation Failed to Reverse the Change in Blood Gases in Chronic OSA

Hypoxia, hypercapnia, and acidosis manifested in the present study, similar to OSA human features, indicating that our chronic OSA model had been successfully established, consistent with a previous study, which reported changes in blood gases at baseline and week 12 (Yang et al., 2019). Additionally, we also found that hypoxia, hypercapnia, and acidosis presented as early as the 4th week, and this abnormal state became serious at the 8th and 12th week regardless of whether the SLGP was ablated. Furthermore, our study also revealed that AF inducibility was significantly decreased under the same hypoxic environment, suggesting that the anti-arrhythmia effect of GP ablation is not mediated by modulating changes in blood gases.

### SLGP Ablation Reversed Atrial Electrical Remodeling in OSA-Induced AF

It has been widely accepted that electrical remodeling contributes to the initiation and maintenance of AF associated with OSA (Oliveira et al., 2009; Latina et al., 2013). Clinical studies have demonstrated that extensive areas of low voltage or electrical silence and conduction delay, which are prone to induce AF, could be observed in patients with OSA (Latina et al., 2013). Several studies reported that ERP was significantly decreased while the WOV was increased in an acute OSA-induced AF model (Yu et al., 2017a,b). A chronic OSA-induced AF model manifested the same result (Zhao et al., 2014). Furthermore, the AF duration and inducibility were also studied at baseline and the 12th week, whereas the changes during this process were not elucidated in detail (Zhao et al., 2014).

Our study first reported consecutive changes in electrophysiological parameters at baseline and at the 4th, 8th, and 12th week. Compared to the baseline, ERP decreased significantly, while the WOV, AF inducibility, and AF duration increased notably at the 4th week. This change tendency deteriorated more at the 8th and 12th week, while SLGP ablation partly or completely reversed these changes within 1 month. Additionally, we also first reported the change tendency of SNRT throughout the experiment and found that SNRT in the OSA group was significantly increased at the 4th week and gradually recovered toward baseline at the 8th and 12th week. No statistically significant difference could be observed in the OSA group and the OSA + GP group at the 12th week.

Previous studies reported that AF from any cause leads to atrial tissue conduction velocity change, thereby increasing the stability of the re-entrant circuits and promoting the progression of AF (van der Velden et al., 2000; Ai et al., 2010; Yang et al., 2019). The conduction velocity was mainly determined by the communication between cells, especially related to Cx40 and Cx43 (Kanagaratnam et al., 2002; Dhein et al., 2014). Yang et al. (2019) demonstrated that the decreased expression level of Cx43 played an important role in the initiation and development of a chronic intermittent hypoxia-induced AF rat model, which was paralleled by another study in which lower expression of Cx43 was also detected in aged rabbit atrial tissue with AF (Yan et al., 2018). However, Zhao et al. (2014) and Sun et al. (2017) reported that Cx40, Cx43, and the Cx40/Cx43 ratio were not significantly changed in a dog model of chronic OSA-induced AF. In our present study, we found that both Cx40 and Cx43 were evidently decreased in the OSA group, and this change could be effectively reversed by SLGP ablation, suggesting that a low expression level of connexin might also be involved in the development of OSA-induced AF.

In short, SLGP ablation could alleviate the deterioration of AF-related electrophysiological parameters and subsequently decrease the occurrence of AF.

### SLGP Ablation Reversed the Echocardiographic Changes in OSA-Induced AF

Previous studies have illustrated that OSA has a strong correlation with LA size, mainly because repetitive swings in intrathoracic pressure are transmitted to the thin-walled atria and lead to atrial dilation and fibrosis, ultimately promoting the occurrence of AF (Orban et al., 2008; Linz et al., 2011a,b; Valenza et al., 2014). Clinical studies have shown that LA enlargement and left ventricular diastolic dysfunction could promote OSA, leading to a predisposition to AF (Latina et al., 2013). Zhao et al. (2014) and Sun et al. (2017) reported that RAd and LAd were enlarged and that LVEF decreased at 3 months in an AF-associated OSA canine model; this change was also observed in our study. Furthermore, we also found that these echocardiographic parameters changed as early as the 4th week and deteriorated more at weeks 8 and 12.

We first reported the change tendency of echocardiography in a 3-month OSA-induced AF canine model and furthermore first investigated whether SLGP ablation could reverse this effect. In addition, our study also showed that effective interventions made at an early stage could result in a better prognosis.

### SLGP Ablation Failed to Reverse Myocardial Tissue Changes in OSA-Induced AF

Myocardial tissue changes in chronic repetitive apneic events play an important role in OSA-induced AF, mainly including atrial enlargement, fibrosis, hypertrophy, myolysis, and other degenerative changes (De Jong et al., 2011). Atrial enlargement and fibrosis are thought to be the two core functions of atrial remodeling (Nattel et al., 2008). Both Zhao et al. (2014) and Sun et al. (2017) reported that atrial apoptosis and fibrosis could be detected in a dog model of chronic OSA-induced AF. In our present study, we also revealed the existence of fibrosis and adipose tissue in the left atrial tissues, while GP ablation failed to reverse those changes in AF associated with OSA within 4 weeks.

In addition, we also first comprehensively reported the changes in myocardial tissue in other chambers. Compared with the CTRL group, considerable fatty infiltration of the LAA, extensive fibrosis of the RAA, and mild fatty infiltration and fibrosis of the LV and RV were present in the OSA + GP group. At the same time, glycogen deposition and cardiomyocyte apoptosis were also observed in myocardial tissues. Previous studies have also shown that chronic intermittent hypoxia (CIH) causes endoplasmic reticulum stress, inflammatory infiltration, increased cardiomyocyte apoptosis, and destruction of mitochondrial structure (Kuang et al., 2004; Zhou et al., 2014). Taken together, these results clearly showed that all of these changes in myocardial structure constitute the arrhythmogenic substrate for OSA-induced AF.

### Change in ANS Activity in the Process of AF Induced by OSA

Some human and animal studies have indicated that sympathovagal imbalance also participates in the onset of OSA-related AF (Linz et al., 2012; Galal, 2017). HRV is known and widely used to evaluate cardiac autonomic activity (Usui and Nishida, 2017). Galal (2017) reported that LF, HF, and the LF/HF ratio were increased in patients with OSA. Yu et al. (2017c) showed that both LF and HF were increased in a 1-h intermittent hypoxia-induced AF dog model, revealing that sympathovagal imbalance contributed to the initiation of AF. However, these parameters in animal studies were acquired under anesthesia, and changes in ANS when awake did not reflect, let alone describe, the dynamic trend of ANS activity.

In our study, we first recorded the dynamic changes in HRV in non-anesthetized dogs throughout the experiment, thus effectively avoiding the effects of anesthetics on the inhibition of sympathetic nerve activity (Liu et al., 2019). In the initial stage of chronic OSA, we did not observe excessive activation of sympathetic nerve activity (SNA) and vagus nerve activity (VNA); with the prolongation of OSA time, both SNA and VNA increased and reached a plateau at the end of the 8th week. At this time, SNA and VNA were the highest, and neural activity tends to decrease. Therefore, we chose this time point to ablate the GP. We found that regardless of whether GP ablation was performed, SNA and VNA were decreased in the OSA group and OSA + GP group, and the scale of decrease was larger in the OSA + GP group. We hypothesized that the autonomic hyperinnervation might trigger an autonomic reflex to reduce the SNA and VNA (especially the SNA) as a protective mechanism after 2 months. We just showed this phenomenon, and more experiments are needed to explore possible mechanisms. In the final stage of OSA, both SNA and VNA in the OSA group decreased, compared with baseline, and still remained active (especially SNA). In addition, the change range of HR and SBP before and after SLGP ablation also supported this opinion (Figures 7A–C). This was partly consistent with Zhao et al. (2014) study, which showed that increasing vagal activation promotes AF inducibility in chronic OSA.

Additionally, we detected that the expression levels of markers of neuronal activity in atrial tissues and the SLGP, not only markers of sympathetic neuron activation (NGF and TH) but also vagus nerve activation markers (CHAT), were notably enhanced. Previous studies (Zhao et al., 2014; Sun et al., 2017) have shown that the expression levels of TH and CHAT-positive fibers were significantly increased in a dog model of chronic OSA-induced AF, indicating that the ANS plays a vital role in OSA-induced neural remodeling. Other studies (Wang et al., 2016; Yu et al., 2016, 2017a,b) have also shown that high expression of NGF promotes cardiac autonomic remodeling and cardiac arrhythmia inducibility, and inhibiting NGF expression could have the effect of treating AF. Our results were consistent with those studies, and we also found that the expression of these neuronal factors was evidently decreased after the SLGP was ablated. Taken together, these results indicated that SLGP ablation suppressed hyperactivity of the ANS and subsequently inhibited AF inducibility.

The imbalanced state of the ANS has been gradually recognized in AF associated with OSA (Carnagarin et al., 2019; Huang et al., 2020). Treatment measures that modulate the ANS, including metoprolol (Li et al., 2015; Sun et al., 2017; Dai et al., 2019) and LLTS (Yu et al., 2017b), could inhibit the inducibility of AF. Recently, atrial GP ablation was also recognized as an important measurement to regulate the ANS. Ghias et al. (2009) and Yu et al. (2017c) showed that GP ablation could effectively suppress the onset of AF in acute simulated OSA-induced AF. However, one more important limitation of those studies was that the chronic effect of GP ablation was not evaluated.

In our study, we confirmed that dozens of neurons were contained in the SLGP, and the myelinated nerve fibers and neurons were obviously destroyed once the SLGP was ablated. Additionally, the dynamic change in HRV and neural markers also showed hyperactivity of the ANS in chronic OSA-induced AF, and the SLGP rebalanced the ANS to a degree. Therefore, we have reason to infer that SLGP ablation suppressed autonomic remodeling and subsequently inhibited the occurrence of AF.

Previous studies reported that mediators of the inflammatory response could alter atrial electrophysiology and structural substrates, thereby leading to increased vulnerability to AF (Hu et al., 2015), and anti-inflammatory measures might be therapeutic for AF patients (Aschar-Sobbi et al., 2015; Yao et al., 2018). In our current study, proteomics analysis also showed inflammatory signaling pathway participation in the progression of OSA-induced AF, and SLGP ablation downregulated inflammatory expression, which was consistent with ameliorated electrophysiological parameters and echocardiography parameters and lower AF inducibility. However, the evident fibrosis in the myocardial tissue was not ameliorated after the GP ablation. To date, just one manuscript by Zhao et al. (2011) has described the relationship between GP ablation and inflammation in AF. They reported that concentrations of TNF-α and IL-6 in the atrium increased significantly 8 weeks post GP ablation, which was a predictor for recurrence of AF after GP ablation. This was contradictory to our research findings. We just demonstrated inflammatory signaling pathway participation in the progression of OSA-induced AF, and SLGP ablation downregulated inflammatory expression. Additional investigation will be required to resolve this discrepancy.

Taken together, all of this evidence suggested that electrical remodeling was independent of atrial structural remodeling, and SLGP ablation could improve the occurrence of electrical remodeling by regulating the abnormal activity of the ANS and inflammation levels, which could suppress the appearance of AF.

### Possible Mechanisms of GP Ablation Inhibiting OSA-Induced AF

**Figure 13:**
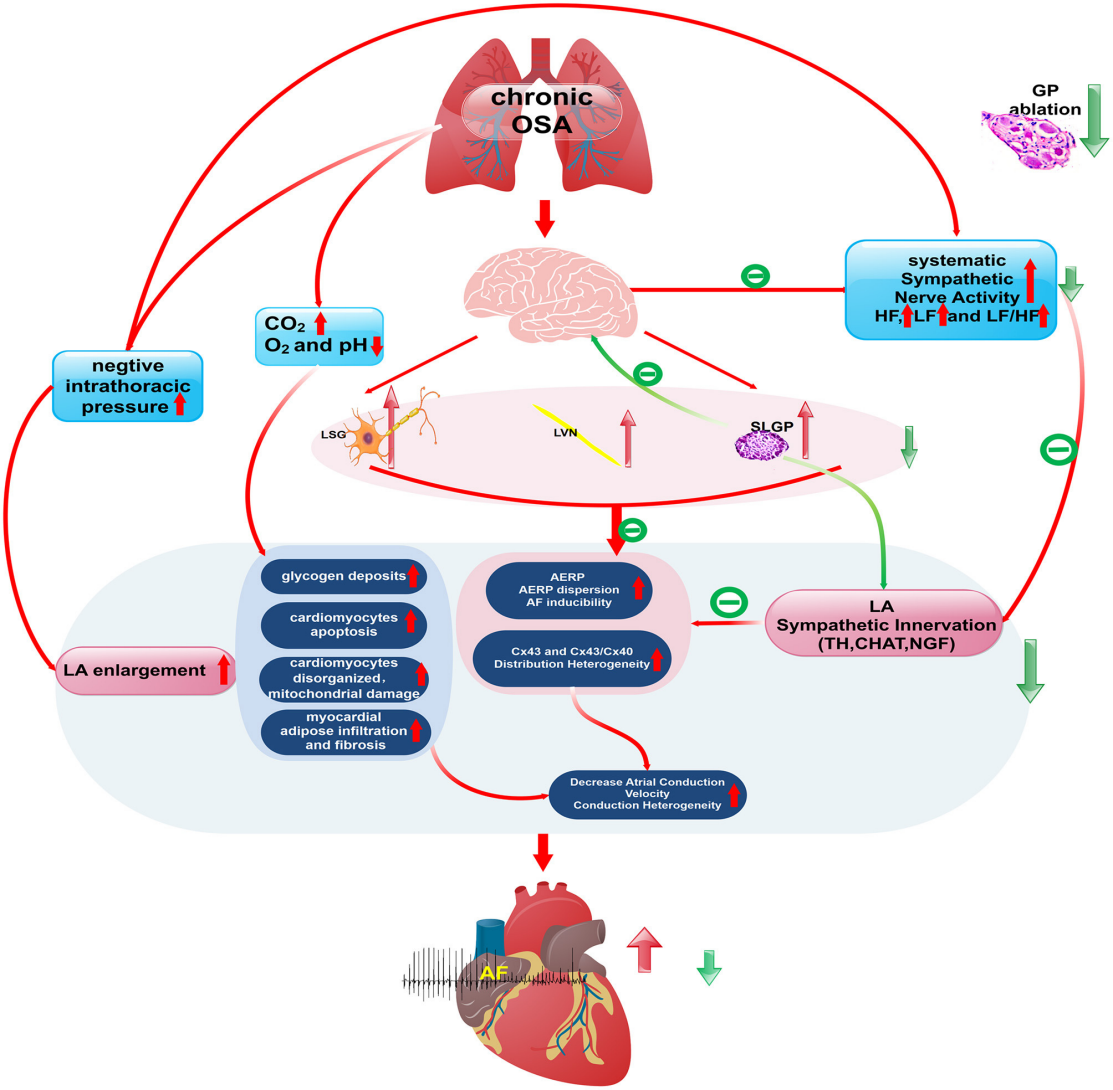
The schematic diagram illustrates the underlying mechanism by which SLGP ablation attenuates chronic OSA-associated AF.

**Figure 14:**
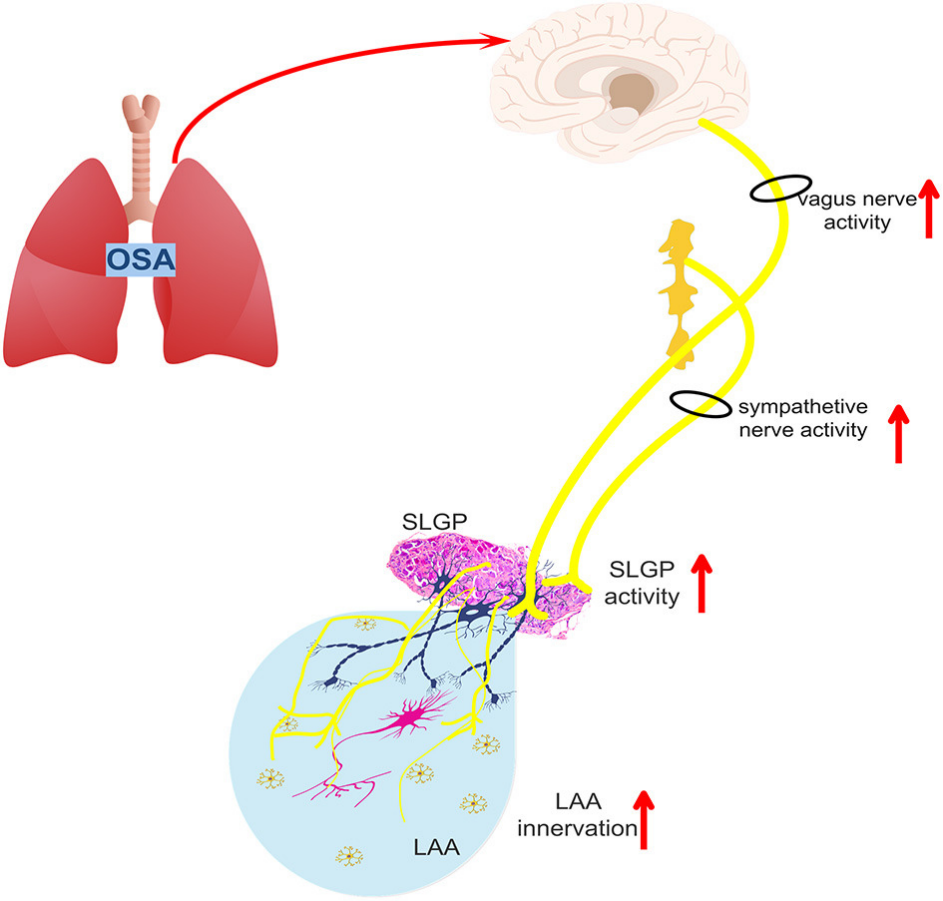
The schematic diagram illustrates the changes in the intrinsic and extrinsic nervous system activity of OSA-related chronic AF.

A schematic diagram illustrates the underlying mechanism by which SLGP ablation attenuates chronic OSA-associated AF (Figures 13, 14). On the one hand, OSA increases afferent signaling to the central nervous system, which leads to overactivity of the intrinsic and extrinsic cardiac autonomic nervous system, thereby increasing the nerve activity of the SLGP, LSG, and LVN, which increases AERP dispersion and AF inducibility and sympathovagal innervation in the LA. On the other hand, chronic repeated OSA leads to hypoxia, negative intrathoracic pressure surges, decreased Cx43 levels, LA enlargement, glycogen deposition, increased necrosis, and fibrosis in cardiomyocytes. All of these changes promote increased conduction heterogeneity and decreased conduction velocity, thereby contributing to the occurrence and maintenance of OSA-induced AF. However, SLGP ablation reduces input to the central nervous system and downregulates the activity of the autonomic nervous system, thereby inhibiting OSA-induced AF.

### Clinical Implications

The long-term treatment effect of SLGP ablation could effectively suppress AF inducibility in chronic OSA in our current study. Previous clinical studies also showed that GP ablation + PVI significantly increased the success rate of AF ablation (Katritsis et al., 2011, 2013; Kampaktsis et al., 2017). Therefore, we hypothesized that SLGP ablation could be considered one of the strategies for chronic OSA-induced AF. Another important aspect was that we observed electrical remodeling and structural remodeling in the early stage of our experiment, so early intervention should be considered in the early progression of OSA to acquire a better prognosis. However, we have only conducted animal studies, so more clinical studies are needed for confirmation.

### Study Limitations

Several limitations must be noted in this study. (1) Due to our technical limitations, we failed to implant a recorder to monitor neural activity in the body in the long term, while we dynamically monitored the change in HRV to reflect the activity of the ANS. (2) Previous studies have confirmed that the bilateral stellate ganglia and vagus nerve have different roles in atrial remodeling. We only focused on the activity of the left stellate ganglion and cervical vagus nerve, which might not fully reflect the extrinsic autonomic nervous system. (3) The third limitation is that the inflammatory signaling pathway was involved in the progression of OSA as determined through proteomics analysis, while the exact pathway has not yet been analyzed and needs further verification.

## Conclusion

Canines with chronic OSA demonstrate significant atrial electrical and structural remodeling characterized by shortening ERP, increased AF inducibility, increased conduction heterogeneity, and sinus node dysfunction. In addition, chamber enlargement, glycogen deposition, increased cardiomyocyte necrosis, and fibrous hyperplasia accompanied by adipose tissue infiltration were noted in the LA. Meanwhile, chronic OSA dramatically increased activities of the intrinsic (SLGP) and extrinsic cardiac ANS (LVN and LSG). All of the factors suggest that the hyperactivity of intrinsic and extrinsic cardiac ANS plays a crucial role in the development of chronic OSA-induced AF. Therefore, SLGP ablation might serve as a potential therapy to treat AF in OSA patients.

## Data Availability Statement

The raw data supporting the conclusions of this article will be made available by the authors, without undue reservation.

## Ethics Statement

The animal study was reviewed and approved by the Committee of the Ethics of Animal Experiments of the First Affiliated Hospital of Xinjiang Medical University.

## Author Contributions

LZ and BT contributed to the funding acquisition, conception, and design of the study. LZ, YG, JX, HS, and KL contributed to the animal experiments. GC, HL, and XZ contributed to the statistical analysis and interpretation. All authors contributed to the writing, critical reading, and approval of the manuscript.

## Conflict of Interest

The authors declare that the research was conducted in the absence of any commercial or financial relationships that could be construed as a potential conflict of interest.
