# Supplementary material for: Ganglionated Plexi Ablation Suppresses Chronic Obstructive Sleep Apnea-Related Atrial Fibrillation by Inhibiting Cardiac Autonomic Hyperactivation
Source: Front Physiol. 2021 Apr 9;12:640295. doi: 10.3389/fphys.2021.640295 (PMC8063039; doi:10.3389/fphys.2021.640295)
Supplement: Supplementary file 1 [file Table_1.DOCX]

The specific genes were listed as follows,

**OSA group vs CTRL group Up-regulated**

| **KEGG pathway** | **-log10(p value)** | **Related genes** |
| --- | --- | --- |
| cfa04260 Cardiac muscle contraction | 3.42 | Q8WN71, E2R8F7, E2RBW4, E2RTH5, P12637, Q28264, F6UPH0 |
| cfa05014 Amyotrophic lateral sclerosis (ALS) | 1.88 | F6UNV5, F6UNP7, F1PGH6 |
| cfa04657 IL-17 signaling pathway | 1.67 | C0LQL0, J9P732, F1P6A2 |
| cfa05144 Malaria | 1.59 | J9JHZ3, P49819, F1PSS2, E2RJE0, E2REA1 |
| cfa05410 Hypertrophic cardiomyopathy (HCM) | 1.36 | Q8WN71, E2R8F7, E2RBW4, E2RFE1 |
| cfa04966 Collecting duct acid secretion | 1.33 | E2R2U7, F1PKH1 |

**OSA+GP vs. OSA Down-regulated**

| **KEGG pathway** | **-log10(p value)** | **Related genes** |
| --- | --- | --- |
| cfa04966 Collecting duct acid secretion | 2.02 | E2R2U7, F1Q1T0 |
| cfa04621 NOD-like receptor signaling pathway | 1.8 | J9PAN9, Q6TN20, J9NSF3 |
| cfa00982 Drug metabolism - cytochrome P450 | 1.7 | E2R5B9, E2RHC8 |
| cfa05323 Rheumatoid arthritis | 1.65 | E2R2U7, F1Q1T0 |
| cfa03460 Fanconi anemia pathway | 1.56 | F1Q0X3 |
| cfa04979 Cholesterol metabolism | 1.48 | J9PAN9, J9NSF3 |
| cfa04216 Ferroptosis | 1.44 | J9PAN9, J9NSF3 |
| cfa00330 Arginine and proline metabolism | 1.4 | E2RAF1, E2RHQ0 |
